# Dietary Supplementation With Hydroxyproline Enhances Growth Performance, Collagen Synthesis and Muscle Quality of *Carassius auratus* Triploid

**DOI:** 10.3389/fphys.2022.913800

**Published:** 2022-06-01

**Authors:** Shenping Cao, Yangbo Xiao, Rong Huang, Dafang Zhao, Wenqian Xu, Shitao Li, Jianzhou Tang, Fufa Qu, Junyan Jin, Shouqi Xie, Zhen Liu

**Affiliations:** ^1^ Hunan Provincial Key Laboratory of Nutrition and Quality Control of Aquatic Animals, Department of Biological and Environmental Engineering, Changsha University, Changsha, China; ^2^ State Key Laboratory of Freshwater Ecology and Biotechnology, Institute of Hydrobiology, Chinese Academy of Sciences, Wuhan, China

**Keywords:** hydroxyproline, growth performance, collagen synthesis, muscle texture, Carassius auratus triploid

## Abstract

An eight-week experiment was undertaken to examine the effect of dietary hydroxyproline (Hyp) supplementation on growth performance, collagen synthesis, muscle quality of an improved triploid crucian carp (*Carassius auratus* Triploid) (ITCC). Six isonitrogenous (340 g/kg diet), isolipidic (60 g/kg diet) and isocaloric (17.80 MJ/kg diet) diets were formulated containing a certain amount of Hyp: 0.09% (the control group), 0.39, 0.76, 1.14, 1.53 and 1.90%. Each diet was randomly assigned to three tanks and each group was fed two times daily until apparent satiation. The results showed that growth performance and feed utilization of ITCC were significantly improved with the dietary Hyp level was increased from 0.09 to 0.76%. Crude protein, threonine and arginine content in the dorsal muscle in 0.76% hydroxyproline group were significantly higher than those in basic diet group (*p* < 0.05). The muscle textural characteristics increased remarkably with the amount of Hyp in the diet rising from 0.09 to 1.53% (*p* < 0.05). Meanwhile, the contents of type I collagen (Col I) and Pyridinium crosslink (PYD) in the muscle of fish were significantly increased by dietary Hyp (*p* < 0.05). The muscle fiber diameter and density of the fish were significantly increased when fed with 0.76% Hyp (*p* < 0.05). Furthermore, dietary supplementation with an appropriate concentration of Hyp substantially increased the expression of genes involved in collagen synthesis (*col1a1*, *col1a2*, *p4hα1*, *p4hβ*, *smad4*, *smad5*, *smad9,* and *tgf-β*) and muscle growth (*igf-1*, *tor*, *myod*, *myf5,* and *myhc*) (*p* < 0.05). In conclusion, dietary supplementation of Hyp can enhance fish growth performance, collagen production, muscle textural characteristics and muscle growth of ITCC. According to the SGR broken-line analysis, the recommended supplementation level of Hyp was 0.74% in the diet for ITCC, corresponding to 2.2% of dietary protein.

## Introduction

Fish meal has always been utilized as a preferred protein source due to its high protein content, excellent essential amino acid composition (EAA) and easy digestibility (Daniel, 2018). However, the limited supply of fish meal around the world has been unable to keep up with the fast development of global aquaculture, resulting in global rising of fish meal price year by year (Fawole et al., 2021). As a result, appropriate plant-derived alternative protein sources have received more and more attention. However, poor growth occurs when some farmed fish are fed diets that include high levels of plant ingredients (Chen et al., 2019b; Liu et al., 2020). Some researchers believed that the deficiency of some amino acids in plant protein feed is an important reason leading to the decrease of growth performance in fish, such as lysine, methionine and hydroxyproline (Hyp) (Hua et al., 2019; Clark et al., 2020).

Hydroxyproline (Hyp), extensively prevalent in marine and animal feedstuffs but mostly absent in plant-based source (Zhang et al., 2015). The hydroxylation of proline residues in protein (mainly collagen) results in the formation of Hyp via prolyl hydroxylase on the endoplasmic reticulum (Li and Wu, 2018). Always, Hyp is known to be a substrate for the production of glucose hydroxyproline, pyruvate and glycine (Li et al., 2009). And it is a conditionally-essential amino acid in aquatic organisms (Wu et al., 2011). So far, some studies in marine carnivorous fish have found that Hyp has different effects on the growth performance of cultured fish. For example, Rong et al. (2020b) discovered that supplementing Hyp in a high plant-protein diet substantially enhanced growth and vertebral development of chu’s croaker (*Nibea coibor*). The growth-related indicators were substantially positively linked with the dietary Hyp levels for Atlantic salmon (*Salmo salar* L.) (Kousoulaki et al., 2009). However, a different result was found in Atlantic salmon that dietary supplementation of different contents of Hyp had no significant effect on growth performance and feed efficiency of fish (Zhang et al., 2013).

Skeletal muscle, composed of muscle fibers and intramuscular connective tissue, is the most palatable portion of fish flesh (Listrat et al., 2016). Collagen, the primary component of intramuscular connective tissue, helps maintain tissue stability and structural integrity and is directly related to muscle hardness and muscle quality (Palka, 1999). In vertebrates, roughly 99.8% of Hyp is located in collagen (Barbul, 2008). Hyp is key to forming triple-helical molecules, which helps to maintain the integrity of collagen fibrils and increases protein heat stability (Gelse et al., 2003; Li and Wu, 2018). Muscle texture is a conventional indicator used in the evaluation of fish flesh quality. Besides collagen concentration, the muscle texture of fish is also remarkably affected by collagen crosslink (Hansen et al., 2007). Research on Atlantic salmon revealed a strong positive correlation between pyridinium crosslink (PYD) content and muscle stiffness (Johnston et al., 2006). Also, the appropriate amount of Hyp can greatly improve the dorsal muscle textural characteristics, and collagen formation was found to be significantly correlated to the levels of PYD, prolyl 4-hydroxylase (P4H) and lysyl hydroxylase (LH) in the muscle of large yellow croaker (*Larimichthys crocea*) (Wei et al., 2016). Furthermore, several investigations in mammals have indicated that the transforming growth factor-beta/SMAD family proteins (TGF-β/Smads) pathway is the primary signaling pathway controlling collagen production, especially the production of type I collagen (Yano et al., 2012; Xu et al., 2016). In contrast, the connection between the TGF-β/Smads pathway and type I collagen in fish is not entirely understood.

Muscle development is a complicated adaptive process that involves both developing new muscle fibers (hyperplasia) and expanding existing muscle fibers (hypertrophy), and it is regulated by a number of elements, including insulin-like growth factor I (IGF-1), myogenic regulators (MRFs) and myosin heavy chain (MyHC) (Rowlerson and Veggetti, 2001). Investigations *in vitro* and *in vivo* have shown that IGF-1, a major regulatory hormone governing vertebrate development, may increase the proliferation and differentiation of myoblasts and induce myotube hypertrophy (Zanou and Gailly, 2013). MRFs are muscle-specific basic helix-loop-helix transcription factors that govern the production of certain proteins throughout the cellular differentiation and determination process (Hernández-Hernández et al., 2017). Myogenic factor 5 (Myf5) and myogenic differentiation antigen (MyoD) as representative transcription factors of MRFs are mainly involved in the initial proliferation process of myoblasts and the directional completion of myogenic cells (Zammit, 2017). Also, MyHC has essential regulatory effects on muscle fiber types and muscle specificity (Song et al., 2020). According to the recent research in turbot (*Scophthalmus maximus*), rich-Hyp fish meal hydrolysate can enhance muscle growth by modulating the expressions of genes related muscle growth, including *myod*, *myf5*, and *mrf4* (Wei et al., 2020). Nevertheless, the precise role of Hyp in regulating muscle development in fish remains unknown.

*Carassius auratus* Triploid is obtained from the crossing of red crucian carp (*Carassius auratus* red var.) × common carp (*Cyprinus carpio* L.) allotetraploid hybrids (♂) with the diploid Japanese crucian carp (♀) (*Carassius auratus cuvieri* T. et S.) (Chen et al., 2009). Because of its advantages of fast growth speed, strong stress tolerance and delicious flesh quality, ITCC is the preferred species of crucian carp in freshwater aquaculture in China (Fu et al., 2021). Up to now, some studies have reported that Hyp can promote growth and improve muscle quality in carnivorous marine fish, whereas Hyp’s potential for the same effect on omnivorous freshwater fish remains unknown. Therefore, ITCC was used as a model in this study to evaluate the dietary requirements of Hyp and its effects on growth performance, collagen production, muscle texture and muscle fiber development.

## Materials and Methods

### Experimental Diets

Table 1 shows the formulation and chemical composition of the experimental diets. Crystalline L-hydroxyproline (Hyp, >99% pure) was purchased from Sigma-Aldrich Co. Ltd. (United States). Six isonitrogenous (340 g/kg diet), isolipidic (60 g/kg diet) and isocaloric (17.80 MJ/kg diet) diets were formulated with graded levels of Hyp: 0% (control group), 0.4%, 0.8%, 1.2%, 1.6 and 2.0%, respectively. Using high-performance liquid chromatography (LC-6AD, Shimadzu Corporation, Japan), the amounts of Hyp in the diet contents were measured, and the final Hyp contents in each diet were 0.09%, 0.39%, 0.76%, 1.14%, 1.53% and 1.90%, respectively. Protein sources included fishmeal, rapeseed meal, cottonseed meal, soybean meal and wheat flour; soybean oil for a lipid source; corn starch and α-starch for the main carbohydrate sources. All feed components were passed through a 40-mesh sieve before thoroughly combined with 10% distilled water for 10 min, then the wet dough was extruded into 2 mm pellets using a laboratory granulator (SZLH200, Jiangsu Zhengchang Group Co. Ltd., China). The feed pellets were gradually dried in the air before being kept in separate sealed plastic bags at 4 C till usage. Table 2 shows the amino acid composition of the experimental diets.

**TABLE 1:** Formulation and chemical composition of the experimental diets for ITCC (% dry matter).

Ingredients	Dietary Hyp Level (%)					
	0.09	0.39	0.76	1.14	1.53	1.90
L-Hydroxyproline^1^	0.00	0.40	0.80	1.20	1.60	2.00
Fish meal^2^	0.50	0.50	0.50	0.50	0.50	0.50
Soybean meal^2^	22.00	22.00	22.00	22.00	22.00	22.00
Rapeseed meal^2^	19.00	19.00	19.00	19.00	19.00	19.00
Cottonseed meal^2^	16.00	16.00	16.00	16.00	16.00	16.00
Wheat flour	13.00	13.00	13.00	13.00	13.00	13.00
Soybean oil	3.70	3.70	3.70	3.70	3.70	3.70
α-starch	4.00	4.00	4.00	4.00	4.00	4.00
Corn starch	8.00	8.00	8.00	8.00	8.00	8.00
Choline chloride	0.50	0.50	0.50	0.50	0.50	0.50
Vitamin premix^3^	1.00	1.00	1.00	1.00	1.00	1.00
Mineral premix^4^	2.00	2.00	2.00	2.00	2.00	2.00
CMC	3.00	3.00	3.00	3.00	3.00	3.00
Ca(H_2_PO_4_)_2_	1.00	1.00	1.00	1.00	1.00	1.00
Cellulose	4.30	4.30	4.30	4.30	4.30	4.30
Alanine^5^	2	1.60	1.20	0.80	0.40	0.00
Hydroxyproline	0.09	0.39	0.76	1.14	1.53	1.90
Crude protein	34.71	34.58	33.99	34.14	34.01	33.74
Crude lipid	5.87	5.82	5.79	6.00	5.96	6.10
Moisture	8.32	8.09	8.12	8.09	7.74	7.60
Ash	8.93	8.88	8.66	8.61	8.68	8.62
Total phosphorus	0.90	0.97	0.96	0.91	0.87	0.94
Total calcium	0.55	0.54	0.55	0.53	0.52	0.50
Gross energy (MJ/kg)	17.87	17.94	18.04	17.72	17.75	18.14

1 L-Hydroxyproline: Purchased from Sigma-Aldrich Co. Ltd (United States).

2 All of these ingredients were supplied by Hunan Zhenghong Science and Technology Develop Co., Ltd., China. Fish meal, crude protein: 68.87%, crude lipid: 10.47%; Soybean meal, crude protein: 48.57%, crude lipid: 1.40%; Rapeseed meal, crude protein: 44.37%, crude lipid: 2.63%; Cottonseed meal, crude protein: 54.60%, crude lipid: 2.02%.

3 Vitamin premix (mg/kg diet): Vitamin B_1_, 20; Vitamin B_2_, 20; Vitamin B_6_, 20; Vitamin B_12_, 0.02; folic acid, 5; calcium pantothenate, 50; inositol, 100; niacin, 100; biotin, 0.1; Vitamin A, 11; Vitamin D, 2; Vitamin E, 50; Vitamin K, 10; Vitamin C, 100; cellulose, 3,412.

4 Mineral premix (mg/kg diet): NaCl, 500.0; MgSO_4_·7H_2_O, 8,155.6; NaH_2_PO_4_·2H_2_O, 12500.0; KH_2_PO_4_, 16000.0; CaHPO_4_·2H_2_O, 7,650.6; FeSO_4_·7H_2_O, 2286.2; C_6_H_10_CaO_6_·5H_2_O, 1750.0; ZnSO_4_·7H_2_O, 178.0; MnSO_4_·H_2_O, 61.4; CuSO_4_·5H_2_O, 15.5; CoSO_4_·7H_2_O, 0.91; KI, 1.5; Na_2_SeO_3_, 0.60; Corn starch, 899.7.

5 Alanine: Purchased from Sigma-Aldrich Co. Ltd (United States).

**TABLE 2:** Amino acid profile of the experimental diets for 8 weeks (% dry matter).

Ingredients	Dietary Hyp Level (%)					
	0.09	0.39	0.76	1.14	1.53	1.90
Essential amino acid						
Threonine	1.01	1.00	0.98	0.98	0.98	0.96
Phenylalanine	1.32	1.30	1.32	1.32	1.31	1.30
Valine	1.17	1.17	1.17	1.15	1.18	1.16
Isoleucine	1.16	1.17	1.16	1.15	1.17	1.16
Leucine	1.99	2.01	1.98	1.99	1.97	1.97
Methionine	0.29	0.29	0.30	0.28	0.29	0.28
Arginine	2.06	2.10	2.09	2.08	2.07	2.05
Lysine	1.46	1.48	1.48	1.45	1.43	1.40
Histidine	0.71	0.71	0.71	0.73	0.70	0.70
Tryptophan	ND^1^	ND	ND	ND	ND	ND
Non-essential amino acid						
Aspartic acid	2.38	2.39	2.39	2.38	2.37	2.37
Serine	1.30	1.33	1.30	1.32	1.30	1.29
Glutamic acid	5.23	5.24	5.26	5.22	5.22	5.20
Glycine	1.17	1.16	1.16	1.14	1.15	1.16
Alanine	2.69	2.30	1.87	1.50	1.12	0.84
Tyrosine	0.74	0.75	0.75	0.75	0.76	0.74
Proline	1.47	1.48	1.46	1.48	1.46	1.45

1 ND: not determined.

### Fish and Feeding Trial

About 1,000 ITCC were purchased from the Fisheries Research Institute of Hunan Province (Changsha, Hunan, China). All the fish were soaked in 4% saline for 20 min before being transferred to the indoor recirculating aquaculture system. Two weeks prior to the formal experiment, all fish were cultured in two cylindrical fiberglass tanks (1,500 L) and fed the compound feed (a combination of six experimental diets) twice daily (9: 00 and 15: 00) to make fish adapt to the experimental feeds and feeding frequency.

Subsequently, all fish were fasted for 24 h before the feeding trial began. Four hundred and fourteen acclimatized fish with the similar size (initial body weight: 15.01 ± 0.05 g) and apparent health were weighed and randomly divided into 18 fiberglass tanks (100 L). With a density of 23 fish per tank, triplicate tanks were randomly assigned to each of the six experimental diets. Fish in all the groups were fed to apparent satiation, twice daily at 9: 00 and 15: 00 h for an 8-weeks feeding trial.

The water flowed into each tank at a constant rate of 1,200 ml min^−1^. During the feeding study, dissolved oxygen and ammonia nitrogen in the experimental system and the experimental tank were determined once a week, the water temperature and pH were measured once a day. The dissolved oxygen was greater than 7.0 mg L^−1^, and the ammonia nitrogen level was below 0.1 mg kg^−1^. The water temperature was kept constant at 27.1 ± 2.9°C, while the pH ranged between 6.5 and 7.0. Furthermore, the photoperiod of this experiment was regularly controlled for a 12 h light-dark cycle (light from 8: 00 to 20: 00).

### Sample Collection

Preceding the feeding trial, 30 fish were randomly selected and separated into three sample groups (10 fish per sample group) for the initial examination of body composition. All fish from each tank were starved for 24 h at the end of the feeding experiment, then netted out and sedated with 50 mg L^−1^ MS-222 (tricaine methane sulfonate, Sigma-Aldrich, United States). Four fish were sampled in triplicate for final body composition analysis. Blood samples were drawn from the caudal vein with heparinized syringes, centrifuged at 3,000 g for 10 min, and kept at −80°C in a refrigerator. Following blood collection, the fish were dissected on ice to obtain the tissues of skin, liver and vertebrae for further detection of Hyp, and the muscle for detection of Hyp, biochemical composition, related enzymes as well as gene expressions analysis. Dorsal muscle of four fish from each tank were dissected on ice for texture analysis, also, dorsal muscle of another two fish were collected and fixed in a 4% paraformaldehyde solution for further histological analysis.

### Biochemical Analysis

The Association of Official Analytical Chemists’ technique was used to assess the approximate composition of the diets, dorsal muscle and whole fish samples (AOAC, 2005). The crude protein was measured using a Kjeltec 8,400 Analyzer Unit machine (FOSS Tecator, Haganas, Sweden), and the crude lipid was determined using the Soxtec ST 243 system (FOSS, Sweden). Moisture was determined by drying at 105°C in an oven to constant weight, and ash content was determined by burning for 3 h in a muffle furnace at 550°C. An automated adiabatic oxygen bomb calorimeter was used to measure the gross energy (Phillipson Microbomb Calorimeter, Gentry Instructions Inc., Aiken, United States), and the dietary and dorsal muscle total amino acid (TAA) was determined using high-performance liquid chromatography (LC-6AD, Shimadzu Corporation, Japan).

### Hydroxyproline Determination

The muscle, skin, liver and vertebrae of ITCC were dissected and diluted in a solution of 0.67% physiological saline. The tissues were then crushed and homogenized using a high-speed tissue grinding device (KZ-II, Wuhan Servicebio technology, China) and centrifuged at 5,000 g for 10 min at 4 C. The Hyp content was determined using the isolated supernatant. The method used to calculate Hyp was modified somewhat from that used by Wei et al. (2016). Aliquots of 1 ml standard Hyp were prepared from a stock solution of Hyp (Sigma-Aldrich Corp., United States). 1 ml standard Hyp (1–50 μg/ml) or 1 ml of hydrolysed tissue sample (plasma, muscle and other tissues) were mixed with 2 ml buffered chloramines T reagent and incubated at room temperature for 30 min. Following incubation, 2 ml perchloric acid (dilute 27 ml 70% perchloric acid to a 100 ml solution) was added to the mixture and incubated for another 5 min at room temperature. The mixture was then heated at 60 C for 20 min with 2 ml of P-DMAB solution. Upon cooling, the absorbance was measured at 560 nm, and the concentration of Hyp was calculated using a standard curve.

### The Type I Collagen, Pyridinium Crosslink, Prolyl 4-Hydroxylase and Lysyl Hydroxylase Assay

The type I collagen (Col I), pyridinium crosslink (PYD), prolyl 4-hydroxylase (P4H) and lysyl hydroxylase (LH) levels in dorsal muscle of ICTT were measured using a biotin double antibody sandwich ELISA kit (Nanjing Jiancheng Bioengineering Institute, Nanjing, Jiangsu, China) according to the manufacturer’s instructions, respectively.

### Muscle Texture Analysis

For the muscle texture examination, three fish from each tank were selected. From the dorsal muscles on both sides above the lateral line and below the dorsal fin of each fish, two pieces fillets was gently removed for texture analysis using a texture analyzer (TMS-PRO, Food Technology Corporation, America). The texture profile analyses (TPA) parameters were built using double compression. The compression ratio of the muscle sample was 60%, and the test speed was 1 mm/s. Each sample’s textural characteristics such as chewiness, springiness, hardness, adhesiveness, cohesiveness and gumminess were calculated via the force-time curve produced using the computer software, Texture Lab Pro (1.18-408, FTC, America).

### Histological Analysis

The histological examination of muscle samples was carried out in accordance with the previously reported technique (Cao et al., 2021). In brief, the muscle samples were cut into rectangular muscle blocks measuring 3 mm × 3 mm × 10 mm along the lateral line, fixed in 4% paraformaldehyde solution for 24 h, and then dried in 70% ethanol. The samples were embedded in paraffin wax after being dehydrated with varying amounts of ethanol and xylene. Sagittal sections with a thickness of 5 μm were taken from each sample, which were then dewaxed with xylene and stained with hematoxylin-eosin (H&E). The image analysis program Image-pro Plus was used to measure the muscle fiber diameter (DI) and density (DO) of sections. For each sample, eight microscopic fields were investigated at random. The contour of 150-250 muscle fibers in each microscopic field was digitized by Image Analysis System, and the diameter of muscle fibers was calculated. The number of fibers per mm^2^ of muscle cross-sectional area was used to calculate fiber density.

### Gene Expression Analysis

The sequences of the primers utilized in the measurement of transcriptional levels of prolyl 4-hydroxylase subunit α-1-like (*p4hα1*), prolyl 4-hydroxylase subunit β (*p4hβ*), collagen type I alpha 1 (*col1a1*), collagen type I alpha 2 (*col1a2*), *tgf-β*, SMAD family member 4 (*smad4*), *smad5*, *smad9*, *igf-1*, target of rapamycin (*tor*), *myod*, *myf5* and *myhc* are shown in Table 3. The PCR primers design used *Carassius auratus* sequences.

**TABLE 3:** Sequences of the primers used for qRT-PCR analysis.

Gene	Acronym	Primer Sequence	Accession No.	Amplicon Size (bp)	Annealing Temp. (°C)
*Collagen type I alpha 1*	*col1a1*	F: TGT​CAC​TGA​GGA​TGG​TTG​CAC	AB275454	83	60
		R: GAC​GGG​ATG​TTT​TCG​TTG​TTT			
*Collagen type I alpha 2*	*col1a2*	F: TGA​GGG​CTA​AGG​ATT​ATG​AGG	AB275455	99	60
		R: GGG​CAG​GGT​TCT​TCT​TTG​AGC			
*Prolyl 4-hydroxylase subunit α-1-like*	*p4hα1*	F: CGT​CTT​CCC​TGG​CAT​CGG​AGT​TG	XM_026207228	93	55
		R: TTA​CCC​AAT​AAC​ACA​GGG​CAT​CC			
*Prolyl 4-hydroxylase subunit β*	*p4hβ*	F: AGG​AGA​GAA​GGA​GAA​CCC​CAA	XM_026213906	133	60
		R: AGC​AAT​AAG​AGA​CTC​CGC​CTG			
*Transforming growth factor-β*	*tgf-β*	F: CCT​GGG​CTG​GAA​GTG​GAT​A	EU086521	190	60
		R: GTA​AAG​GAT​GGG​CAG​TGG​G			
*SMAD family member 4*	*smad4*	F: TGGCTGGTCGTAAAGGAT	XM_026274091	152	60
		R: CTC​GTA​ATG​GTA​AGG​GTT​CA			
*SMAD family member 5*	*smad5*	F: CGA​GGT​GTG​CGA​GTA​TCC​GTT	XM_026281121	169	55
		R: ATT​GTG​ACT​CAG​GTT​GCG​AAA			
*SMAD family member 9*	*smad9*	F: GCA​CTC​CAC​TAC​ATC​CAT​CAC	XM_026274041	93	55
		R: TTT​TCC​TCC​TCA​TCT​CCT​TGT			
*Insulin-like growth factor 1*	*igf-1*	F: AGC​GTG​TCT​ACA​AGC​TCC​G	KF813006	175	60
		R: GGA​TGT​CTA​GCG​GTC​ATT​TCT			
*Target of rapamycin*	*tor*	F: TTG​ATG​GCA​CGG​TGT​TTC​CTA​A	KF772613	195	60
		R: GCC​CTG​GTT​CTG​GTG​CTT​GTA​G			
*Myogenic differentiation antigen*	*myod*	F: ACCAGAGGCTGCCCAAAG	KP715154	123	60
		R: AGT​CTC​CGC​TGT​AAT​GTT​CC			
*Myogenic factor 5*	*myf5*	F: GTTTGAGGCACTACGGCG	KP715152	192	60
		R: CTT​TCA​GAA​CAG​CTT​GAG​GAA​G			
*Myosin heavy chain*	*myhc*	F: GTG​CTT​GAC​ATT​GCT​GGG​TT	XM_026260328	143	60
		R: ATG​CCT​TCT​TTC​TTG​TAT​TCC​T			
*β-actin*	*β-actin*	F: TTG​AGC​AGG​AGA​TGG​GAA​CCG	AB039726.2	115	60
		R: AGA​GCC​TCA​GGG​CAA​CGG​AAA			

TRIzol reagent was used to extract total RNA from muscle tissues (Invitrogen Life Technologies). Next, 1.2% agarose gel electrophoresis was used to evaluate the integrity of the RNA, and spectrophotometry was used to determine the quantity of RNA (BioPhotometer, Eppendorf). The PrimeScrip RT kit was then used to reverse-transcribe total RNA into complementary DNA (cDNA) (Takara, Japan). The obtained cDNA was stored at −20°C. A CFX96 Real-time PCR Detection System (Bio-Rad, United States) was utilized to perform a quantitative real-time polymerase chain reaction (qRT-PCR). Then, 16 μl volume was used for the amplification, which included 8 μl 2 × SYBR Premix ExTaq polymerase (Takara), 1 μl cDNA, 0.64 μl forward and reverse primers, 0.32 μl ROX Reference Dye (20 μM) and 5.4 μl ddH_2_O. The following were the cycling conditions for the qRT-PCR reaction: Pre-incubation at 95°C for 3 min, then 40 cycles of 95°C for 10 s, annealing temperature (about 60°C, Table 3) for 20 s, and 72°C for 10 s. In order to identify the relative expression levels of the target genes, *β-actin* was utilized as an internal reference. The comparative CT technique (2^−ΔΔCt^ method) was used to do relative quantification of qRT-PCR (Livak and Schmittgen, 2001).

### Calculations and Statistical Analysis

At the end of experiment, the growth parameters were calculated as follows:

Weight gain rate (WGR, %) = (total final body weight—total initial body weight)/total initial body weight × 100.

Specific growth rate (SGR, % day^−1^) = [(ln FBW—ln IBW)/days] × 100.

Feed efficiency (FE, %) = (total final body weight—total initial body weight)/total dry feed intake × 100.

Feeding rate (FR, % body weight day^−1^) = (total dry feed intake)/[days × (total final body weight + total initial body weight)/2] × 100.

Protein efficiency ratio (PER, %) = (total final body weight—total initial body weight)/total protein intake × 100.

Nitrogen retention efficiency (NRE, %) = (total final whole fish protein content—total initial whole fish protein content)/total protein intake × 100.

Total nitrogen waste output (TNW, g kg^−1^ weight gain) = (total protein intake × (100—NRE))/((total final body weight—total initial body weight) × 6.25) × 100.

Survival rate (SR, %) = (final fish number/initial fish number) × 100.

All data in Figures and Tables were performed as mean ± standard error (SE). SPSS 19.0 was used to conduct the statistical analysis (SPSS Inc., Chicago, IL, United States). Growth, biochemical composition, enzymatic activities, muscle texture, histological examination and gene expression underwent a one-way analysis of variance (ANOVA) followed by Duncan’s multiple range test. When *p* < 0.05, differences were considered significant. The optimal supplemented level of Hyp in the diet on SGR was calculated via broken-line regression analysis (y = L−U × (R−x)) in Origin 7.5 (Origin Software, CA, United States).

## Results

### Growth Performance

**FIGURE 1:**
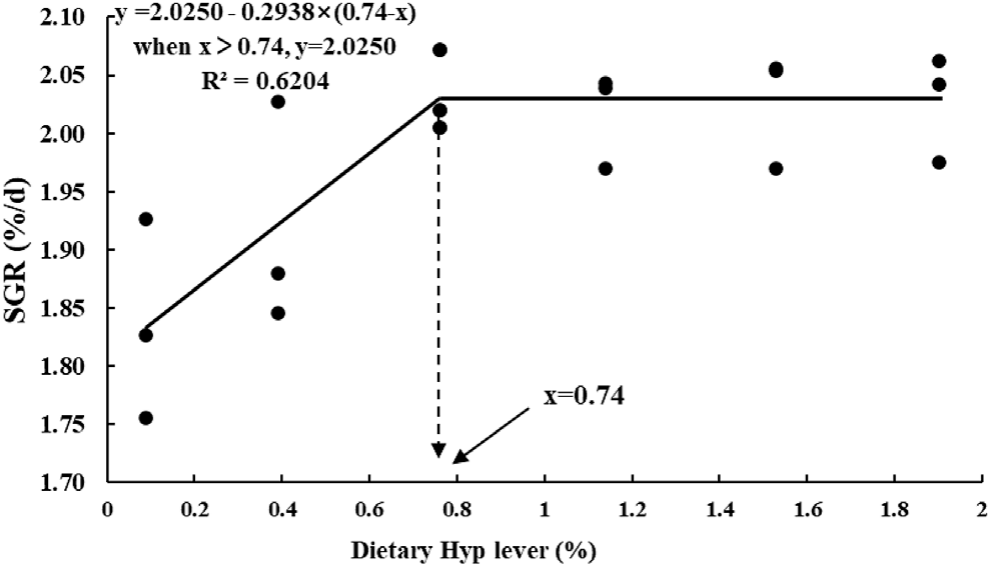
Broken-line regression analysis between SGR and dietary Hyp level in ITCC fed with different experimental diets for 8 weeks. The breakpoint of the broken-line is 0.74% of dry diet.

Table 4 shows the growth performance and feed utilization of fish fed diets with different levels of Hyp. Final body weight (FBW), FE, WGR and SGR were significantly increased with increasing of dietary Hyp level from 0.09 to 0.76% (*p* < 0.05), however, with dietary Hyp supplemented levels further increased, FBW, FE, WGR and SGR remained largely unchanged. In contrast, FR exhibited an inverse relationship with SGR. According to the broken-line regression analysis, the association between SGR and dietary Hyp level was: y = 2.0250–0.2938 × (0.74−x) (*R* ^2^ = 0.6204, Figure 1). For the growth of ITCC, the optimal Hyp content in the diet was established at 0.74%. In terms of protein utilization, PER and NRE showed a similar trend to SGR with the gradual increase of Hyp in the diets. TNW was lowest in the group of fish that were fed the 0.76% Hyp supplemented diet, followed by 0.39, 1.14, 1.53, 1.90%, and highest in the control group.

**TABLE 4:** Growth, feed utilization and survival rate of ITCC fed with the experimental diets containing different levels of Hyp for 8 weeks.

Items	Dietary Hyp Level (%)					
	0.09	0.39	0.76	1.14	1.53	1.90
IBW (g)	14.99 ± 0.04	15.01 ± 0.02	14.99 ± 0.05	15.05 ± 0.02	15.03 ± 0.02	15.00 ± 0.02
FBW (g)	41.94 ± 1.07^a^	43.95 ± 1.32^ab^	46.79 ± 0.62^b^	46.58 ± 0.58^b^	46.76 ± 0.72^b^	46.68 ± 0.69^b^
WGR (%)	179.76 ± 7.78^a^	192.92 ± 9.22^ab^	212.10 ± 3.54^b^	209.49 ± 4.10^b^	211.16 ± 4.88^b^	211.12 ± 4.55^b^
SGR (%/d)	1.84 ± 0.05^a^	1.92 ± 0.06^ab^	2.03 ± 0.02^b^	2.02 ± 0.02^b^	2.03 ± 0.03^b^	2.03 ± 0.02^b^
FE (%)	44.60 ± 1.73^a^	47.21 ± 2.60^a^	52.68 ± 0.86^b^	52.43 ± 1.05^b^	53.90 ± 1.22^b^	52.62 ± 1.04^b^
FR (%)	3.54 ± 0.06^b^	3.43 ± 0.08^b^	3.26 ± 0.03^a^	3.25 ± 0.04^a^	3.18 ± 0.04^a^	3.25 ± 0.03^a^
PER (%)	128.49 ± 4.97^a^	141.04 ± 10.45^ab^	154.97 ± 2.53^bc^	153.57 ± 3.08^bc^	158.47 ± 3.59^c^	155.99 ± 3.08^c^
NRE (%)	22.71 ± 0.85^a^	23.40 ± 1.99^a^	27.05 ± 1.09^b^	25.63 ± 0.84^ab^	25.67 ± 0.48^ab^	25.35 ± 1.18^ab^
TNW (g/kg WG)	45.57 ± 0.92^b^	43.12 ± 2.65^b^	38.77 ± 0.71^a^	39.66 ± 0.87^a^	38.56 ± 0.75^a^	39.33 ± 0.97^a^
SR (%)	100.00 ± 0.00	100.00 ± 0.00	100.00 ± 0.00	100.00 ± 0.00	100.00 ± 0.00	100.00 ± 0.00

Values are Means ± SE (n = 3); a, b, c mean values in the same row with different superscript letters are significantly different (*p* < 0.05); Absence of letters indicates no significant difference between treatments.

IBW: initial body weight. FBW: final body weight. WGR: weight gain rate. SGR: specific growth rate. FE: feed efficiency. FR: feeding rate. PER: protein efficiency ratio. NRE: nitrogen retention efficiency. TNW: total nitrogen waste output. SR: survival rate.

### Chemical Composition of Whole Fish and Dorsal Muscle

The ash level of whole fish samples treated with 0.39% Hyp was considerably higher than in the control group (*p* < 0.05) (Table 5). Initially, the content of crude protein was significantly enhanced when dietary Hyp increased from 0.09 to 0.76% but reduced as the Hyp level in the diet was further increased. Meanwhile, dietary Hyp level had no effect on crude protein, crude lipid and moisture content in whole fish samples (*p* > 0.05). No significant changes in moisture and ash were detected in dorsal muscle samples (*p* > 0.05). In addition, with the rising level of dietary Hyp supplementation, the content of most essential amino acids in dorsal muscle first increased and then decreased (Table 6). In particular, the levels of threonine and arginine increased significantly as the dietary Hyp supplemental level was raised from 0.09 to 0.76% but decreased gradually with additional Hyp supplemented in the diets (*p* < 0.05).

**TABLE 5:** Whole body and dorsal muscle compositions of ITCC fed with different experimental diets for 8 weeks.

Items	Dietary Hyp Level (%)					
	0.09	0.39	0.76	1.14	1.53	1.90
Whole body composition (% fresh weight)						
Crude protein	15.72 ± 0.33	15.98 ± 0.11	16.35 ± 0.71	16.59 ± 0.59	16.01 ± 0.16	15.68 ± 0.06
Crude lipid	11.04 ± 0.5	11.18 ± 2.01	12.1 ± 0.55	12.17 ± 1.16	13.06 ± 0.91	13.57 ± 1.34
Ash	2.85 ± 0.16^a^	2.47 ± 0.12^b^	2.52 ± 0.07^ab^	2.71 ± 0.08^ab^	2.63 ± 0.05^ab^	2.58 ± 0.08^ab^
Moisture	69.85 ± 0.24	68.15 ± 0.46	68.22 ± 0.38	68.18 ± 0.79	67.94 ± 0.97	67.3 ± 1.22
Dorsal muscles composition (% fresh weight)						
Crude protein	17.06 ± 0.12^a^	17.69 ± 0.11^ab^	18.55 ± 0.73^b^	17.79 ± 0.19^ab^	17.57 ± 0.28^ab^	17.26 ± 0.55^ab^
Crude lipid	1.32 ± 0.07	1.24 ± 0.07	1.28 ± 0.06	1.30 ± 0.04	1.25 ± 0.08	1.20 ± 0.04
Ash	1.44 ± 0.06	1.38 ± 0.06	1.42 ± 0.04	1.38 ± 0.12	1.39 ± 0.04	1.51 ± 0.01
Moisture	75.65 ± 0.09	76.23 ± 0.42	76.00 ± 0.13	76.55 ± 0.57	75.92 ± 0.96	75.69 ± 0.75

Values are Means ± SE (n = 3); a, b, c mean values in the same row with different superscript letters are significantly different (*p* < 0.05); Absence of letters indicates no significant difference between treatments.

**TABLE 6:** Amino acid profile in dorsal muscle of ITCC fed with different experimental diets for 8 weeks (% dry matter).

Amino Acids	Dietary Hyp Level (%)					
	0.09	0.39	0.76	1.14	1.53	1.90
Essential amino acid						
Threonine	2.62 ± 0.09^a^	2.86 ± 0.15^ab^	3.01 ± 0.08^b^	2.87 ± 0.03^ab^	2.89 ± 0.03^ab^	2.65 ± 0.04^a^
Phenylalanine	2.95 ± 0.11	3.16 ± 0.18	3.29 ± 0.12	3.24 ± 0.03	3.23 ± 0.09	2.95 ± 0.08
Valine	2.98 ± 0.11	3.13 ± 0.15	3.27 ± 0.10	3.19 ± 0.01	3.24 ± 0.05	2.97 ± 0.07
Isoleucine	3.03 ± 0.10	3.21 ± 0.16	3.37 ± 0.11	3.29 ± 0.02	3.31 ± 0.07	3.02 ± 0.08
Leucine	5.36 ± 0.18	5.68 ± 0.29	5.96 ± 0.18	5.80 ± 0.04	5.84 ± 0.11	5.34 ± 0.20
Methionine	1.72 ± 0.03	1.96 ± 0.11	2.05 ± 0.06	1.97 ± 0.02	1.97 ± 0.03	1.56 ± 0.27
Arginine	3.67 ± 0.13^a^	3.94 ± 0.22^ab^	4.12 ± 0.12^b^	4.05 ± 0.04^ab^	4.02 ± 0.09^ab^	3.71 ± 0.06^ab^
Lysine	6.01 ± 0.29	7.07 ± 0.43	7.19 ± 0.41	7.23 ± 0.10	6.95 ± 0.37	6.17 ± 0.41
Histidine	3.21 ± 0.14^ab^	3.42 ± 0.16^ab^	3.51 ± 0.10^ab^	3.54 ± 0.08^ab^	3.57 ± 0.09^b^	3.16 ± 0.05^a^
Tryptophan	ND^1^	ND	ND	ND	ND	ND
Non-essential amino acid						
Aspartic acid	6.84 ± 0.25	7.23 ± 0.40	7.58 ± 0.24	7.35 ± 0.07	7.40 ± 0.08	6.85 ± 0.32
Serine	2.74 ± 0.09	2.86 ± 0.15	3.00 ± 0.08	2.91 ± 0.02	2.95 ± 0.05	2.72 ± 0.04
Glutamic acid	9.74 ± 0.33	10.19 ± 0.58	10.75 ± 0.33	10.41 ± 0.10	10.54 ± 0.10	9.71 ± 0.48
Glycine	2.93 ± 0.06	2.95 ± 0.14	3.07 ± 0.05	3.05 ± 0.06	3.06 ± 0.03	2.93 ± 0.01
Alanine	3.27 ± 0.10	3.45 ± 0.20	3.63 ± 0.10	3.53 ± 0.04	3.53 ± 0.05	3.26 ± 0.08
Tyrosine	2.15 ± 0.08	2.27 ± 0.11	2.38 ± 0.07	2.31 ± 0.01	2.33 ± 0.05	2.16 ± 0.02
Proline	2.10 ± 0.05	2.09 ± 0.08	2.16 ± 0.04	2.15 ± 0.03	2.18 ± 0.03	2.06 ± 0.04

1 ND: not determined.

Values are Means ± SE (n = 3); a, b, c mean values in the same row with different superscript letters are significantly different (*p* < 0.05); Absence of letters indicates no significant difference between treatments.

### Muscle Texture

Table 7 displays the muscle texture results. Dietary Hyp inclusion substantially enhanced hardness, and the maximum value of hardness determined to be 4.30 in the 1.90% Hyp supplemented group (*p* < 0.05). Meanwhile, chewiness, springiness and gumminess were increased remarkably as the level of Hyp in the diets were raised from 0.09 to 1.53%, whereas these indices were decreased when the level of Hyp in the diet was 1.90%. And among the six treatments, there was no significant change in adhesiveness and cohesiveness (*p* > 0.05).

**TABLE 7:** Muscle texture of ITCC fed with different experimental diets for 8 weeks.

Items	Dietary Hyp Level (%)					
	0.09	0.39	0.76	1.14	1.53	1.9
Hardness (N)	3.67 ± 0.17^a^	4.11 ± 0.24^ab^	3.89 ± 0.08^ab^	4.12 ± 0.11^ab^	4.16 ± 0.08^b^	4.30 ± 0.11^b^
Springiness (mm)	0.51 ± 0.04^a^	0.54 ± 0.03^ab^	0.63 ± 0.04^b^	0.54 ± 0.02^ab^	0.64 ± 0.05^b^	0.60 ± 0.04^ab^
Chewiness (mJ)	0.60 ± 0.07^a^	0.60 ± 0.08^a^	0.68 ± 0.09^ab^	0.55 ± 0.06^a^	0.93 ± 0.13^b^	0.67 ± 0.10^ab^
Adhesiveness (N*mm)	0.031 ± 0.004	0.032 ± 0.005	0.037 ± 0.004	0.031 ± 0.003	0.027 ± 0.003	0.031 ± 0.005
Cohesiveness (%)	0.38 ± 0.03	0.37 ± 0.03	0.39 ± 0.02	0.36 ± 0.02	0.43 ± 0.03	0.37 ± 0.03
Gumminess (g*mm)	1.04 ± 0.12^a^	1.16 ± 0.14^ab^	1.20 ± 0.11^ab^	1.10 ± 0.10^a^	1.54 ± 0.14^b^	1.13 ± 0.16^a^

Values are Means ± SE (n = 9); a, b mean values in the same row with different superscript letters are significantly different (*p* < 0.05); Absence of letters indicates no significant difference between treatments.

### The Hydroxyproline Content in Different Tissues

Dietary Hyp levels had a significant effect on the amount of Hyp in different tissues of ITCC (*p* < 0.05) (Table 8). The Hyp contents in muscle and liver were substantially higher in the 0.76% Hyp supplemented group when compared to the non-Hyp group (*p* < 0.05). In addition, the levels of Hyp in the skin, vertebrae and plasma were all considerably higher following a gradient of increased Hyp level in feed, and the 1.90% group reached the maximum value (*p* < 0.05).

**TABLE 8:** The hydroxyproline content in different tissues of ITCC fed with different experimental diets for 8 weeks.

Tissues	Dietary Hyp Level (%)					
	0.09	0.39	0.76	1.14	1.53	1.90
Muscle (mg/g)	0.91 ± 0.10^a^	1.37 ± 0.01^ab^	1.64 ± 0.19^b^	1.41 ± 0.16^ab^	1.46 ± 0.17^ab^	1.44 ± 0.24^ab^
Skin (mg/g)	8.77 ± 0.93^a^	10.33 ± 0.31^ab^	13.15 ± 1.10^bc^	11.98 ± 0.94^abc^	9.66 ± 0.94^ab^	13.99 ± 1.52^c^
Liver (μg/g)	256.88 ± 5.69^a^	553.99 ± 72.59^ab^	671.96 ± 103.44^b^	567.93 ± 195.71^ab^	430.59 ± 46.70^ab^	456.84 ± 73.89^ab^
Vertebra (mg/g)	7.39 ± 0.17^a^	7.62 ± 0.49^a^	8.45 ± 0.73^ab^	9.47 ± 0.89^ab^	9.04 ± 1.18^ab^	10.5 ± 0.40^b^
Plasma (mg/ml)	100.00 ± 9.62^a^	119.84 ± 7.57^ab^	124.60 ± 12.01^ab^	141.27 ± 10.68^bc^	142.86 ± 4.76^bc^	170.63 ± 9.36^c^

Values are Means ± SE (n = 6); a, b, c mean values in the same row with different superscript letters are significantly different (*p* < 0.05); Absence of letters indicates no significant difference between treatments.

### Col I, Pyridinium Crosslink and Relative Enzymatic Activities in Muscle

Dietary Hyp levels ranging from 1.53 to 1.90% substantially enhanced the contents of Col I and PYD in muscle (*p* < 0.05) (Table 9). Furthermore, the P4H activity of fish fed diets containing 1.14%, 1.53%, and 1.90% Hyp was substantially greater than the control group (*p* < 0.05). LH activity gradually increased with increasing dietary Hyp level, and the greatest value of LH was achieved in the 1.90% Hyp supplemented group (*p* < 0.05).

**TABLE 9:** The type I collagen (Col I) content, pyridinium crosslink (PYD) content, prolyl 4-hydroxylase (P4H) and lysyl hydroxylase (LH) activity in muscle of ITCC fed with different experimental diets for 8 weeks.

Items	Dietary Hyp Level (%)					
	0.09	0.39	0.76	1.14	1.53	1.90
Col I (ng/mg prot)	4.88 ± 0.02^a^	5.53 ± 0.36^ab^	5.34 ± 0.45^ab^	5.13 ± 0.24^ab^	6.02 ± 0.27^b^	5.98 ± 0.27^b^
PYD (ng/mg prot)	25.74 ± 1.06^a^	28.76 ± 0.60^ab^	26.91 ± 1.62^ab^	26.73 ± 1.64^ab^	30.37 ± 1.17^b^	30.79 ± 1.18^b^
P4H (ng/mg prot)	0.66 ± 0.03^a^	0.74 ± 0.03^ab^	0.75 ± 0.04^ab^	0.83 ± 0.05^b^	0.81 ± 0.07^b^	0.84 ± 0.05^b^
LH (ng/mg prot)	0.136 ± 0.005^a^	0.142 ± 0.003^ab^	0.157 ± 0.007^ab^	0.154 ± 0.005^ab^	0.154 ± 0.004^ab^	0.161 ± 0.013^b^

Values are Means ± SE (n = 6); a, b mean values in the same row with different superscript letters are significantly different (*p* < 0.05); Absence of letters indicates no significant difference between treatments.

### Histological Analysis

**FIGURE 2:**
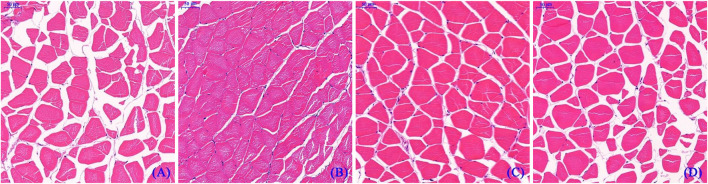
Microstructure of dorsal muscle of ITCC in cross-section. Bars represent 50 μ; **(A–D)** represent different dorsal muscle from the 0.09, 0.76, 1.14 and 1.90% of Hyp supplemental groups, respectively.

Figure 2 depicts the HE staining results of the dorsal muscle in a cross-section, and Table 10 shows the effects of dietary Hyp on muscle fiber diameter (DI) and density (DE) in ITCC. The density of muscle fiber was greater in fish fed Hyp-supplemented diets than in the control group (*p* < 0.05). Furthermore, muscle fiber diameter rose progressively as the dietary Hyp supplemental level was raised from 0.09 to 0.76% but thereafter declined with dietary Hyp further increased.

**TABLE 10:** The muscle fiber diameter (DI) and density (DE) of ITCC fed with different experimental diets for 8 weeks.

Items	Dietary Hyp Level (%)					
	0.09	0.39	0.76	1.14	1.53	1.90
DI (μm)	47.49 ± 1.94^a^	55.84 ± 1.98^b^	61.48 ± 1.36^c^	58.12 ± 1.13^bc^	56.39 ± 1.29^b^	49.14 ± 1.79^a^
DE (n/mm^2^)	158.67 ± 6.44^a^	175.00 ± 7.09^ab^	221.67 ± 18.00^b^	218.00 ± 19.08^b^	184.67 ± 19.38^ab^	167.67 ± 6.06^a^

Values are Means ± SE; a, b, c mean values in the same row with different superscript letters are significantly different (*p* < 0.05); Absence of letters indicates no significant difference between treatments.

### Gene Expression

**FIGURE 3:**
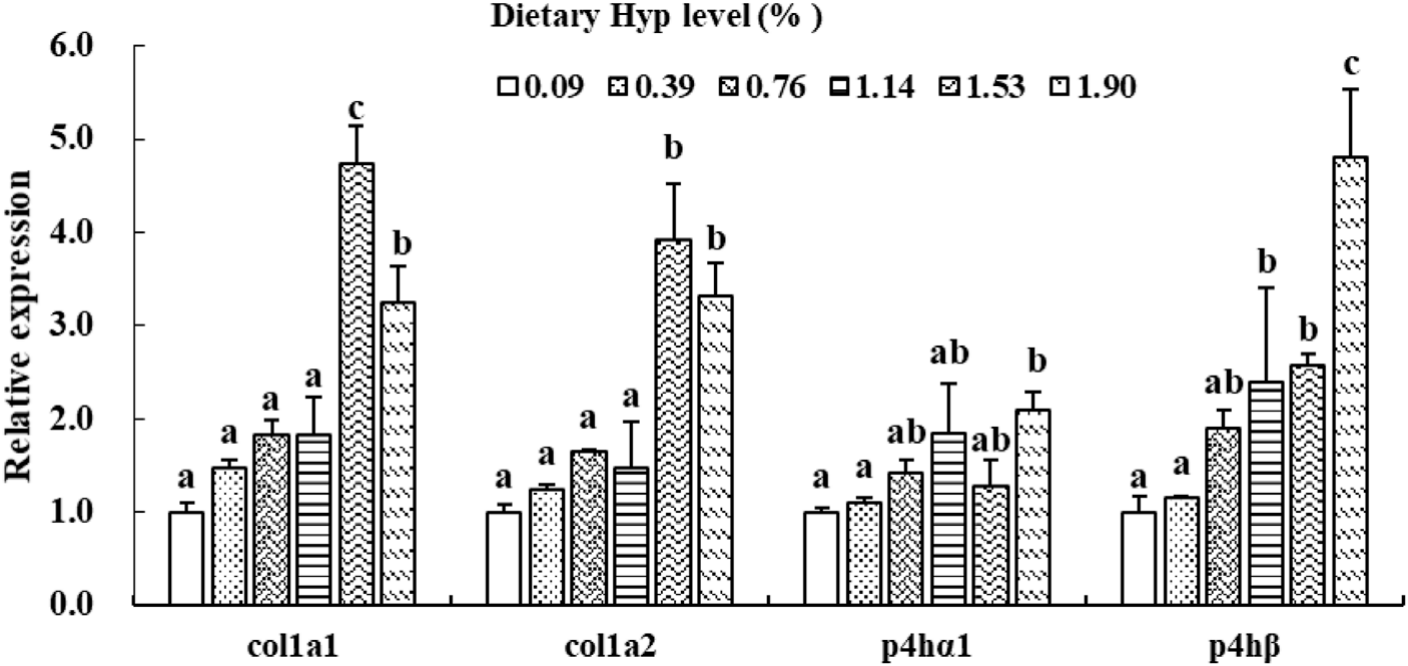
Relative expressions of *col1a1*, *col1a2*, *p4hα1* and *p4hβ* in muscle of ITCC fed with different experimental diets for 8 weeks. Data are present as Means ± SE (n = 6). Different lowercase letters above the same group of bars represent significant difference between the corresponding groups (*p* < 0.05).

**FIGURE 4:**
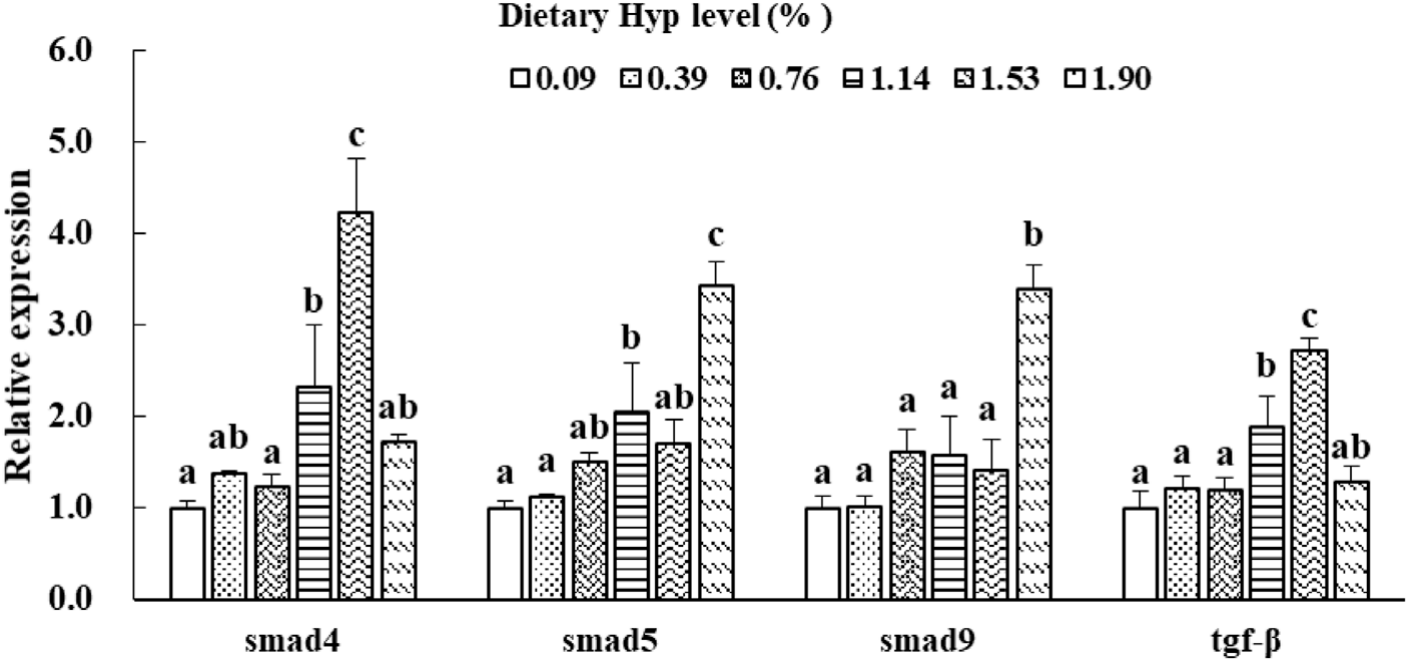
Relative expressions of *smad4*, *smad5*, *smad9* and *tgf-β* in muscle of ITCC fed with different experimental diets for 8 weeks. Data are present as Means ± SE (n = 6). Different lowercase letters above the same group of bars represent significant difference between the corresponding groups (*p* < 0.05).

**FIGURE 5:**
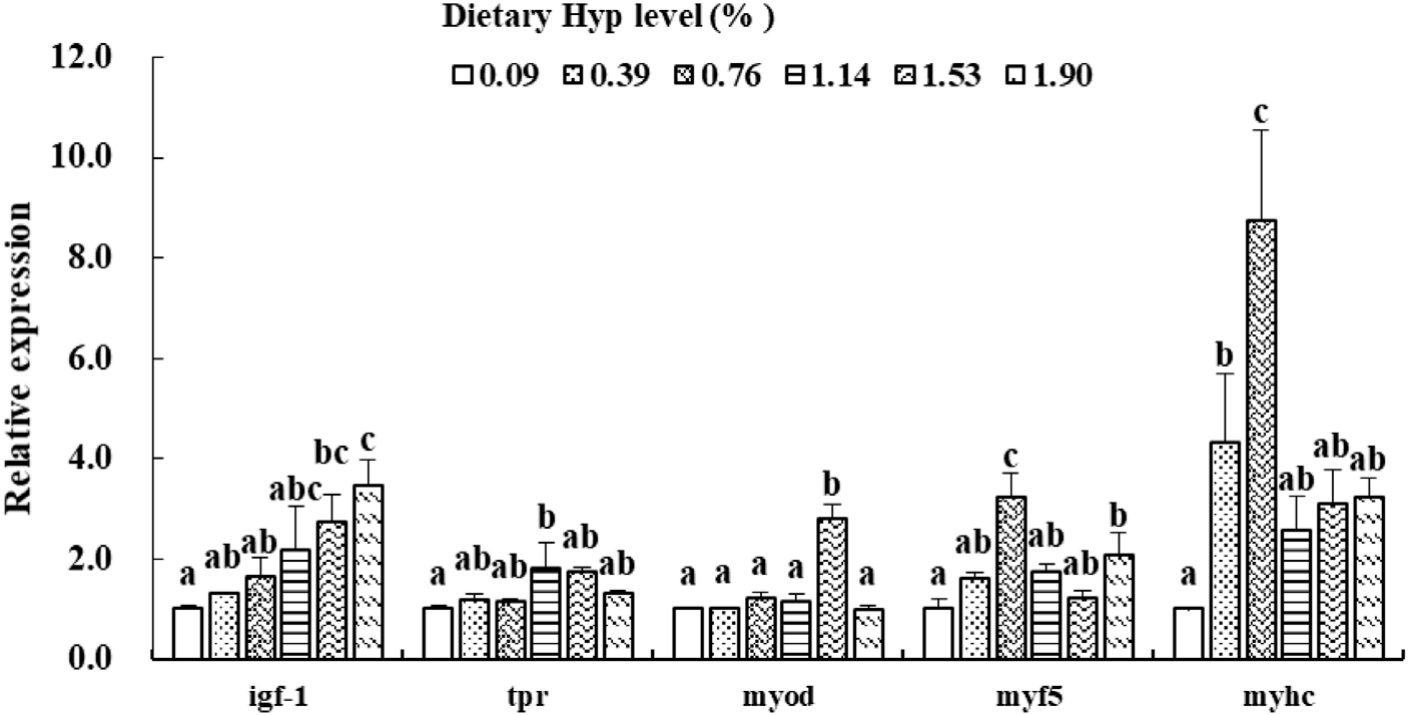
Relative expressions of *igf-1*, *tor*, *myod*, *myf5* and *myhc* in muscle of ITCC fed with different experimental diets for 8 weeks. Data are present as Means ± SE (n = 6). Different lowercase letters above the same group of bars represent significant difference between the corresponding groups (*p* < 0.05).

As shown in Figures 3, 4, the *col1a1*, *col1a2*, *p4hα1*, *p4hβ*, *smad4*, *smad5*, *samd9* and *tgf-β* levels gradually increased with enhancing of Hyp levels in the diets (*p* < 0.05). The maximum expression levels of *col1a1*, *col1a2*, *smad4* and *tgf-β* appeared in the 1.53% Hyp supplemented group, and the maximum levels of *p4hα1*, *p4hβ*, *smad5* and *samd9* appeared in the 1.90% Hyp group. The expression levels of genes related to muscle growth and development are shown in Figure 5. Dietary Hyp levels substantially increased the transcriptional level of *igf-1* expression (*p* < 0.05). Muscle mRNA levels of *tor* were significantly higher in fish fed the 1.14% Hyp diet than fish fed the control diet, whereas *myod* mRNA levels were higher in fish fed the 1.53% Hyp diet. In addition, *myf5* and *myhc* mRNA expressions significantly rose with increasing Hyp level up to 0.76%, and decreased thereafter (*p* < 0.05).

## Discussion

Hydroxyproline (Hyp), as a conditionally necessary amino acid (Li et al., 2009), is plentiful in fish meal but lacking in plant protein sources commonly used in aquatic feed (Li et al., 2011). As a result, the possible negative consequences of Hyp deficiency should be addressed in diets high in plant-based feedstuff. Previous researches have shown that supplementation of Hyp in the diets can significantly increase the growth performance and feed utilization of some marine and carnivorous fish, including large yellow croaker (Wei et al., 2016), Atlantic salmon (Aksnes et al., 2008) and Chinese perch (*Siniperca chuatsi*) (Feng et al., 2022). In the present study, WGR, SGR and FE of ITCC were remarkably enhanced by increasing dietary Hyp level to 0.76%. These results agree with the finding that WG and SGR in the spotted drum (*Nibea diacanthus*) increased significantly when dietary Hyp content rose from 0 to 1%, but stayed stable when Hyp in the diet rose from 1 to 2.5% (Rong et al., 2020a). Rong et al. (2020a) revealed that enhancement of Hyp on growth and protein synthesis ability might be closely related to the significant upregulation of the TOR signaling pathway and upstream GH-IGF-1 axis. However, in some other studies, dietary addition of Hyp showed no significant influence on the growth performance of Atlantic salmon (Albrektsen et al., 2010) and turbot (Zhang et al., 2015). These findings showed that the effect of an Hyp-containing diet regimen on fish growth might be related to aquatic animal size and species, culture environment, and other feed nutrient content. Furthermore, when the dietary Hyp supplemented level was increased to more than 0.76% in the current study, fish growth did not further improve. The data indicates that the optimal requirement of Hyp in ITCC is about 0.76%, and the extra Hyp cannot be used for the growth of fish. The broken-line regression analysis of SGR and dietary Hyp level indicated that 0.74% dietary Hyp content was optimum for the growth of ITCC.

In general, the weight gain of fish is mainly due to muscular protein and lipid accretion (Tu et al., 2015). In this study, the utilization efficiency of feed protein (PER, NRE, and TNW) and the crude protein of dorsal muscle in the fish fed diet containing 0.76% Hyp was considerably greater than in fish provided a control diet. Crude protein of the whole body also showed a gradual upward trend with more Hyp in the diet (*p* > 0.05). The result was consistent with the previous researches in large yellow croaker and spotted drum, which found dietary protein utilization, crude protein in the whole body, dorsal muscle and swim bladder were all significantly elevated by dietary Hyp (Wei et al., 2016; Rong et al., 2020a). These phenomena indicated that dietary inclusion of appropriate Hyp might help with protein synthesis in the tissues of fish, contributing to the growth performance of fish. Protein deposition in fish is, in essence, amino acid deposition (Kaushik and Seiliez, 2010). In the current study, the contents of different amino acids in the dorsal muscle of ITCC exhibited a certain degree of increase under the influence of dietary Hyp, particularly threonine and arginine, which both reached maximum levels when 0.76% Hyp was supplemented. Nutrition research has found that the balance of dietary essential amino acids in vertebrates is key to the efficient absorption and utilization of amino acids (Heger and Frydrych, 2019). Some studies in fish have found that supplementation of amino acids lacking in diets can effectively improve the utilization efficiency of inferior diets and increase the deposition levels of protein and amino acids (Clark et al., 2020; Zhao et al., 2020). In this study, Hyp was the essential amino acid for fish fed diets high in plant-based ingredients. Dietary inclusion of appropriate amounts of Hyp improved dietary amino acid balance, promoting protein synthesis and growth of ITCC.

According to the study of Pinilla-Tenas et al. (2003), Hyp is likely to be absorbed and transported directly to tissues without dihydroxylation. In the current study, Hyp was significantly accumulated in the tissues (muscle, skin, liver, vertebrae and plasma) of ITCC with the increase of dietary Hyp. Similar findings were obtained in Chinese perch and Atlantic salmon, where dietary Hyp supplementation dramatically raised total Hyp content in plasma, liver, vertebrae and muscle (Aksnes et al., 2008; Feng et al., 2022). Hyp content is always regarded as a significant indication of collagen concentration since it is nearly almost dense in collagen. In particular, as dietary Hyp increased, we saw a substantial rise in type I collagen (Col I) content in muscle, which was consistent with a progressive increase in muscular Hyp. The texture mechanical properties (hardness, springiness, chewiness, adhesiveness, cohesiveness and gumminess) are conventional indicators widely used to evaluate fish flesh quality (Hyldig and Nielsen, 2001; Moreno et al., 2012). In this study, the level of Hyp in the diet was increased from 0.09 to 1.53%, which resulted in substantial muscular improvement in hardness, springiness, chewiness and gumminess. Similarly, a sufficient amount of dietary Hyp supplementation has been shown to improve the muscle collagen content and texture of chu’s croaker (Rong et al., 2020b), turbot (Liu et al., 2014) and Atlantic salmon (Albrektsen et al., 2010), suggesting that supplementation of Hyp aids in the improvement of ITCC muscle quality, most likely via raising the concentration of muscular collagen.

Collagen production involves several post-translational and co-translational modifications of polypeptide chains. P4H is the primary form of prolyl hydroxylase (PHD), an important collagen-specific enzyme that catalyzes the hydroxylation of proline residues to 4-Hyp (Gorres and Raines, 2010). *P4hα1* and *p4hβ*, two subunits of *p4h* gene identified in vertebrates, are the pivotal genes involved in regulating collagen maturation and secretion (Sekiya et al., 2005). We observed that P4H activity, *p4hα1* and *p4hβ* expression in fish muscle of specimens fed high-level Hyp were considerably higher than in control group fish. This result demonstrates that including adequate Hyp in the diet can stimulate collagen synthesis in fish muscle. Previous research has revealed that the muscle texture of fish is altered by both collagen crosslinking and concentration (Li et al., 2005). Hagen et al. (2007) discovered that the cross-linking process is critical for collagen hardness and strength and that PYD was the most essential parameter influencing fish muscle texture. Furthermore, the endogenous enzyme LH is known to be involved in the production of collagen crosslinks, which can catalyze lysine hydroxylation to hydroxylysine in collagen molecules (Yamauchi and Shiiba, 2002). In the present study, we observed significantly enhanced PYD content and LH activity in muscle with the inclusion of adequate dietary Hyp, which is similar with the results in large yellow croaker (Wei et al., 2016) and rainbow trout (*Oncorhynchus mykiss*) (Johnsen et al., 2011). These results showed evidence that dietary supplementation of Hyp improved the muscle texture properties of ITCC by promoting the biosynthesis of collagen in muscle and increasing the content of collagen crosslink.

Type 1 collagen (Col I), a primary constituent of collagen in fish muscle connective tissue, consists of one α2 (I) chain and two α1 (I) chains that undergo *col1a2* and *col1a1* gene regulation (Sato et al., 1997; Büttner et al., 2004). In this study, significant higher *col1a1* and *col1a2* genes expressions and Col I content in muscle were observed in the 1.53–1.90% Hyp supplemented groups. This result was consistent with a study of Chinese perch in which diets were supplemented with 1.92-2.37% of Hyp, the relative expression levels of *col1a1* and *col1a2* genes in muscle were significantly upregulated (Feng et al., 2022). Similarly, research on model mice suggests that Hyp content in the skin correlated positively with the expressions of connective tissue growth factor (*ctgf*) and *col1a1* mRNA (Liao et al., 2006). Moreover, *in vitro* research has showed that *tgf-β* significantly increased *col1a1* mRNA in mouse mesangial cells (Kato et al., 2007). TGF-β signal transduction occurs mainly through the activation of receptor substrate Smads protein pathway to achieve a signal from the cell membrane receptor into the nucleus (Moustakas et al., 2001). Smad4 is a critical regulator in the TGF-β/Smads signal pathway and is also key to the control of type I collagen expression in animals (Xia et al., 2021). Smad5 and Smad9 are two representative subtypes of receptor-activated Smad (R-Smad) (Kim and Kim, 2014). In the present study, the gene expressions of *smad4*, *smad5*, *smad9* and *tgf-β* were increased by dietary Hyp inclusion, especially in the high Hyp supplemented group. A similar result was found that feeding grass carp (*Ctenopharyngodon idella*) with faba bean significantly enhanced the mRNA and protein expressions of TGF-β and Smad4, as well as the expression of type I collagen (*col1a1* and *col1a2*) (Yu et al., 2019). These studies indicated that dietary supplementation of appropriate Hyp could increase substrates of collagen synthesis in tissues, thus up-regulating the expression of related genes and promoting Type 1 collagen biosynthesis and accumulation in fish. However, with the increase of Hyp in the diet, collagen content in fish muscle increased while *p4h* (*p4hα1*, *p4hα2,* and *p4hα3*), *col1a1*, and *col1a2* genes down-regulated in turbot (Zhang et al., 2013) and *Nibea diacanthus* (Rong et al., 2019). These investigators hypothesized that consuming Hyp supplements improves collagen levels in fish muscle by preventing collagen degradation rather than boosting collagen synthesis. Therefore, more research is needed to determine the mechanism of dietary Hyp on collagen production in fish muscle.

As it accounts for 40–60% of body weight, skeletal muscle is an integral part of the fish carcass. In fish, muscle fiber is the basic unit of skeletal muscle, representing the level of muscle development (Xu et al., 2019). Variations in dorsal muscle fiber diameter and density provide guidance for understanding of fish muscle growth and development. The current findings for the first time reveal that dietary supplemented Hyp dramatically enhanced fiber diameter and density in the dorsal muscle of fish, indicating that Hyp has positive effects on muscle growth. IGFs influence the development of fish skeletal muscle and can induce the proliferation and differentiation of myoblasts (Fuentes et al., 2013). As a crucial regulatory gene downstream of the GH-IGF-I growth axis, *tor* is required for promoting translation initiation and increasing muscle protein synthesis (Wullschleger et al., 2006). This study revealed that dietary supplementation with adequate Hyp dramatically up-regulated the relative expressions of *igf-1* and *tor* genes in muscle. Similarly, Rong et al. (2020a) demonstrated that optimal dietary Hyp significantly elevated the *tor* expression in the muscle, liver and swim bladder of the juvenile spotted drum. Zhao et al. (2020) suggested that leucine, another essential amino acid, improved muscle protein synthesis in hybrid catfish (*Pelteobagrus vachelli* × *Leiocassis longirostris*) by up-regulating *igf-1* and *igf-1r* mRNA levels. Studies on mammals found that Hyp levels are highly consistent with *igf-1* relative expression (Chen et al., 2019a; Xiao et al., 2020). These results partially explain the mechanism of dietary Hyp increasing muscle growth of fish. Moreover, fish muscle fiber is formed by the proliferation and differentiation of activated satellite cells, a process that is also influenced by a number of myogenic regulatory elements (Rescan, 2005). *Myod* and *myf5* play an important regulatory role in the activation and proliferation of satellite cells (Zammit, 2017). *Myhc* affects fish muscle growth by promoting the proliferation and hypertrophy of muscle fibers (Song et al., 2020). The present studies showed that the appropriate level of dietary Hyp upregulated the gene expressions level of *myod*, *myf5* and *myhc* in fish muscle. This result was consistent with reports on turbot and Atlantic salmon that showed dietary Hyp-rich fish meal hydrolysate and fish bone hydrolysate improved muscle growth and upregulated the expressions of muscle *myod*, *myf5,* and *mrf4* (Albrektsen et al., 2018; Wei et al., 2020). These studies indicated that muscle growth and differentiation were at least partially positively regulated by intramuscular *myod*, *myf5* and *myhc* genes in ITCC. Recently, some growing evidences suggest that essential amino acids play a regulatory role in fish muscle development. For instance, methionine, histidine and lysine have been proved to affect muscle hyperplasia and up-regulate *myog* and *myod* gene expression in fish (Childress et al., 2016; Michelato et al., 2017; Alami-Durante et al., 2018). Hyp, as a conditionally essential amino acid, is presumed to have a similar regulating impact on fish muscle growth. Notwithstanding, additional research is needed to elucidate how dietary Hyp affects the expression of genes associated with muscle growth in fish.

## Conclusion

This study demonstrates that dietary supplementation of 0.76% or more Hyp can significantly improve growth and feed utilization of ITCC. Dietary inclusion of Hyp improved Hyp accumulation in various tissues, muscle textural characteristics, collagen content in muscle and expressions of collagen synthesis-related genes. In addition, an appropriate level of Hyp in the diet significantly elevated protein synthesis in muscle by regulating muscle growth-related gene expression. According to the SGR broken-line analysis, the optimal level of Hyp in the diet for ITCC was estimated to be 0.74%.

## Data Availability

The original contributions presented in the study are included in the article/supplementary material, further inquiries can be directed to the corresponding author.
